# A synthetic peptide encoded by a random DNA sequence inhibits discrete red light responses

**DOI:** 10.1002/pld3.170

**Published:** 2019-10-14

**Authors:** Tautvydas Shuipys, Raquel F. Carvalho, Maureen A. Clancy, Zhilong Bao, Kevin M. Folta

**Affiliations:** ^1^ Genetics and Genomics Graduate Program University of Florida Gainesville FL USA; ^2^ Horticultural Sciences Department University of Florida Gainesville FL USA; ^3^ Plant Molecular and Cellular Biology Program University of Florida Gainesville FL USA

**Keywords:** *Arabidopsis thaliana*, peptides, photomorphogenesis, random DNA sequence, red light, synthetic biology

## Abstract

We have identified a synthetic peptide that interrupts discrete aspects of seedling development under red light. Previous reports have demonstrated that plants transformed with random DNA sequences produce synthetic peptides that affect plant biology. In this report, one specific peptide is characterized that inhibits discrete aspects of red light‐mediated photomorphogenic development in *Arabidopsis thaliana* . Seedlings expressing the PEP6‐32 peptide presented longer hypocotyls and diminished cotyledon expansion when grown under red light. Other red light‐mediated seedling processes such as induction of *Lhcb* (*cab*) transcripts or loss of vertical growth remained unaffected. Long‐term responses to red light in PEP6‐32 expressing plants, such as repression of flowering time, did not show defects in red light signaling or integration. A synthesized peptide applied exogenously induced the long‐hypocotyl phenotype under red light in non‐transformed seedlings. The results indicate that the PEP6‐32 peptide causes discrete cell expansion abnormalities during early seedling development in red light that mimic weak *phyB* alleles, yet only in some aspects of seedling photomorphogenesis. The findings demonstrate that new chemistries derived from random peptide expression can modulate specific facets of plant growth and development.

## 1 INTRODUCTION

There is significant interest in identifying new molecules that modulate plant growth and development, as well as protect them from biotic and abiotic stress. Efforts in chemical genomics have identified novel compounds with specific interactions in the plant, including flowering regulators (Fiers et al., 2017), exocytosis inhibitors (Zhang et al., 2016), and activators of hormonal signaling (De Rybel et al., 2009). Synthetic molecules that serendipitously excite specific biological processes provide a means to create phenotypes in plants that bypass barriers of genetic redundancy or lethality (Hagihara, Yamada, Itami, & Torii, 2019). These approaches drive discovery of new connections between chemistry and biology with the goal of devising new strategies for plant protection, agricultural productivity, or directed synthesis of particular secondary metabolites.

Another approach sought to identify novel regulators in populations of transgenic plants where each plant expressed a unique peptide encoded by randomized DNA information (Bao, Clancy, Carvalho, Elliott, & Folta, 2017). *Arabidopsis thaliana* plants were transformed with libraries of constructs bearing the random DNA sequence, flanked by start and stop codons, and driven by the constitutive CaMV35S promoter. Populations of transformed plants presented a surprisingly frequent number of reproducible phenotypes, apparently due to expression of the random DNA sequence, or potentially its encoding RNA. The results of Bao et al. (2017), identified a new candidate peptide that showed conditional lethality and another (noted as PEP6‐32) that affected seedling growth in red light, but not under blue or far‐red wavelengths.

Red light is sensed through the light sensor family known as the phytochromes (reviewed in Quail, 2002; Xu, Paik, Zhu, & Huq, 2015). The phytochrome B sensor (phyB) has a central role in red light responses (Parks & Spalding, 1999; Reed, Nagpal, Poole, Furuya, & Chory, 1993). In the developing seedling, phyB effects hypocotyl elongation, cotyledon expansion, orientation to the gravitational vector, chloroplast development, and gene expression (Chen & Chory, 2012; Somers, Sharrock, Tepperman, & Quail, 1991; Tepperman et al., 2004; Whitelam, Patel, & Devlin, 1998). Later in development phyB is central in response to shaded environments (Robson, Whitelam, & Smith, 1993; Whitelam & Smith, 1991) and has an important role in the transition from vegetative growth to flowering (Valverde et al., 2004).

The present work attempts to describe where the PEP6‐32 peptide interacts with plant biology. The central question is whether the peptide is interfering with phyB signaling directly or whether it is interfering with an aspect of seedling development only observed under red light illumination. Arabidopsis has been extensively characterized for early light responses and hosts a great array of genetic tools. Therefore, it is possible to test the hypothesis that the PEP6‐32 peptide is specifically interfering with phyB‐regulated light signaling by characterizing a suite of phyB‐related responses.

This report presents a comprehensive physiological characterization of the effect of the PEP6‐32 peptide on red light signaling and response. Such careful characterization is necessary before there can be meaningful efforts to identify the precise mechanism of light‐signal attenuation, as identifying interacting molecules requires understanding precisely where and when the interaction is taking place. The work also tests the possibility of using this synthetic sequence as an exogenous growth regulator in interfering with red light response.

## 2 MATERIALS AND METHODS

### 2.1 Plant materials

The *PEP6‐32* peptide‐expressing plants were grown from seeds generated previously as described in Bao et al. (2017). Corresponding non‐transformed controls were obtained from segregating sibling seedlings not expressing a coincident GFP marker, or for some tests, Col‐0 was used as a non‐transformed control. The phyB‐5 mutant has been previously described (Reed et al., 1993).

### 2.2 Hypocotyl elongation assay

Seeds were surface sterilized with consecutive washes of 70% ethanol for 3 min, 10% bleach for 15 min, and then five rinses with sterile water. Seeds were then planted on minimal media (1 mM CaCl_2_, 1 mM KCl with 0.8% phytoagar) on square (100 × 100 mm) plates. Once planted, plates were stratified in darkness at 4°C for 4 days. The plates were then given 5 hr of white light (~130 μmol m^−2^ s^−1^) to induce germination and placed vertically in custom‐built LED light chambers under constant red light (30 μmol m^−2^ s^−1^), constant blue light (10 μmol m^−2^ s^−1^), or complete darkness for 96 hr. Fluence rate measurements were taken using a Li‐Cor LI‐250 light meter. After the light treatment, the plates were scanned on a flatbed scanner and ImageJ was used to measure individual hypocotyls against an imaged standard. Between 9 and 17 seedlings were imaged per genotype in each experiment with three independent replicates.

### 2.3 Seedling directional growth

IC Measure software (The Imaging Source, LLC) was used to measure the plant angle of growth from images taken during three previous hypocotyl elongation assays. Multiple measurements were taken, and an average calculated for plants that switched major direction of growth mid development. The average angle of growth was calculated by converting each individual angle to 0–180° where 0° is vertical against the direction of gravity and 180° is straight down, in the direction of gravity.

### 2.4 Cotyledon expansion assay

Seeds were surface sterilized by spraying them in 70% ethanol on paper and allowing to dry in a laminar airflow hood. Seeds from two independent transgenic lines were then plated on a deep Petri dishes (100 × 25 mm) containing 0.5x MS basal medium (cat. RPI #M10200) and 0.8% phytoagar with each line covering one half of the plate. Afterward, the plates were stratified in darkness at 4°C for 4 days. The plates were then given 5 hr of white light (~130 μmol m^−2^ s^−1^) to induce germination and placed horizontally in light chambers at  100 μmol m^−2^ s^−1^ white light, 30 μmol m^−2^ s^−1^ red, or 10 μmol m^−2^ s^−1^ blue under 16‐hr light conditions for 7 days. After treatment, seedlings were randomly chosen and cotyledons were removed under a dissecting microscope and imaged. ImageJ software was used to measure the area of each cotyledon. Between 72 and 83 cotyledons were analyzed per genotype.

### 2.5 Time to flower assay

Seeds were surface sterilized and planted on square plates containing 0.5× MS basal media, solidified with 0.8% phytoagar. The plates were stratified in darkness at 4°C for 4 days and placed vertically under white light (~130 μmol m^−2^ s^−1^). At 7 days after germination, uniform seedlings were transferred to soil. In addition, the *PEP6‐32* seedlings were screened to confirm GFP expression (verifying transgene presence) before planting. Each 10‐cm pot containing soilless mix (Fafard 2P) contained two independent lines with three plants per line, and each line was replicated in three pots. The plants were then grown in a growth chamber (Percival Model E36L) under long‐day (16‐hr day, 8‐hr night) conditions at a temperature of 21°C. Flowering was scored as when the inflorescence was 1 cm in length or greater.

Flowering time was also examined under enriched red light conditions. Ambient light spectra were measured in a fluorescent growth chamber using a StellarNet Inc spectroradiometer (Model EPP2000) and SpectraWiz software. The conditions were approximated using LED‐based illumination to best match the fluorescent light spectrum. Two additional light chambers were set to the same spectrum, and the amount of red (660nm) light was increased to 220% and 325% while decreasing 590nm light to maintain the same overall PAR (see Figure S1). Plants were prepared using the same method as above except the plates were grown in the respective LED light chamber (control white, red 220%, or red 325%) prior to transplanting into pots.

### 2.6 Gene expression

Red light induces transcripts from specific genes in etiolated seedlings rapidly after treatment. To measure the expression of the red light‐regulated genes, seeds were surface sterilized and planted on small Petri dishes (60 × 15 mm) containing 0.5 × MS media. The plates were stratified in darkness at 4°C for 4 days and placed under white light (~130 μmol m^−2^ s^−1^) for 5 hr to induce germination. Afterward, the plates were placed in darkness at room temperature for four days. The etiolated seedlings were then exposed to 8 μmol m^−2^ s^−1^ of red light for 1 hr in the LED light chamber or placed into complete darkness. Following treatment, plants were immediately frozen in liquid nitrogen. The shoots were ground to a fine powder with cold mortar and pestle, and RNA was isolated using a Qiagen RNeasy kit. The RNA was treated with Promega RQ1 DNase and reverse transcribed into cDNA using the Improm‐II Reverse Transcription kit (Promega). Steady‐state transcript levels were then measured using the cDNA template in quantitative PCR (qPCR) reactions with SYBR Green reagents. Ubiquitin family protein (*UFP*) was used as the endogenous control. Table S1 shows sequences of *CAB2* and *LHCB1.5* primers used. The fold change in gene expression reported is an average of three RT‐qPCR replicates.

For peptide expression analysis, mature leaves of plants were harvested and flash frozen. Next, 0.1 g of tissue was used in the protocol described above. Peptide expression was then compared relative to Line 1. The expression levels reported are an average of two RT‐qPCR runs.

### 2.7 Exogenous application of synthesized peptide

The PEP6‐32 peptide (MACPASVSVC) was synthesized commercially and dissolved in a buffer containing 1 mM CaCl_2_, 1 mM KCl, and 10 mM HEPES. The peptide concentration was determined with a spectrophotometer. Seeds were surface sterilized and stored in 1.5‐ml Eppendorf tubes with 1 ml of sterile water for stratification in darkness at 4°C for 3 days. Afterward, the seeds were planted on square plates containing minimal media with added peptide at a final concentration of 1 μM or on control plates with an equivalent amount of buffer without peptide. The plates were then placed under white light (~130 μmol m^−2^ s^−1^) for 5 hr. After which, they were transferred to LED light chambers set to 1, 5, 10, 30, or 100 μmol m^−2^ s^−1^ of constant red light for 96 hr or kept dark as a control. Hypocotyl lengths were measured as described above.

### 2.8 Statistical analysis

A one‐way ANOVA with post hoc Tukey HSD test was implemented for hypocotyl elongation tests. The Mann–Whitney *U* test (two‐tailed) was used for other statistical analyses.

## 3 RESULTS

### 3.1 *PEP6‐32* expressing seedlings exhibit elongated hypocotyls under red light

The initial characterization of the *PEP6‐32* line revealed that the seedlings exhibited decreased hypocotyl growth inhibition under red light, but not under far‐red light or dark‐growth conditions (Bao et al., 2017). Furthermore, there was only a slight difference in hypocotyl length under low fluence blue light (≤0.5 μmol m^−2^ s^−1^) that was absent under higher fluence rates. These results suggested that the peptide might be interfering with light sensing or response through phytochrome B (Neff and Chory, 1998). To further explore this possibility, transgenic *PEP6‐32* overexpression lines were compared with non‐transformed controls and *phyB‐5* mutant seedlings. These *phyB* mutants contain a substitution changing W552 to STOP in the coding sequence, resulting in a non‐functional, truncated PHYB protein (Reed et al., 1993). The original mutant (Ler ecotype) was crossed against a *phyA* mutant (Col‐0 ecotype), and then, *phyB* mutants were identified in segregating F2 progeny under red light. These *phyB* mutants were then backcrossed to Col‐0 (as described in Wang et al., 2013) leading to isolation of the *phyB‐5* null‐allele in the appropriate ecotype for use in these experiments.

**Figure 1:**
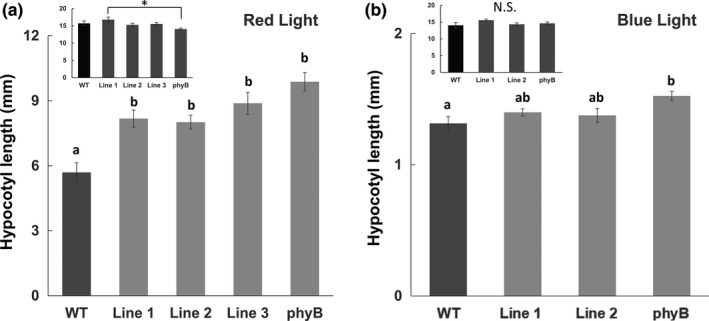
Expression of the *PEP6‐32* peptide results in seedlings with longer hypocotyls than WT and similar to *phyB* mutants under red light. Hypocotyl length of non‐transformed control Col‐0 (WT), independent transformed lines expressing the PEP6‐32 peptide (Lines 1–3), and *phyB* seedlings under 30 μmol m^−2^ s^−1^ red light (a) or 10 μmol m^−2^ s^−1^ blue light (b) after 96 hr. Insets for both panels show the hypocotyl lengths of corresponding seedlings in complete darkness. *N* = 9–10 for Lines 1–3, *N* = 16 for *phyB*, and *N* = 17 for WT. Lower case letters above the bars indicate grouping after one‐way ANOVA with post hoc Tukey HSD test. For insets, the asterisk indicates statistical difference after one‐way ANOVA with post hoc Tukey HSD test; *p* < .05. Error bars indicate standard error of the mean

Non‐transformed Col‐0 seedlings, several independent transformants expressing the *PEP6‐32* peptide, and *phyB* were grown vertically in light chambers under constant red light (30 μmol m^−2^ s^−1^), constant blue light (10 μmol m^−2^ s^−1^), or complete darkness for 96 hr. Under red light, the peptide‐expressing plants and *phyB* mutants showed significantly less hypocotyl growth inhibition compared with non‐transformed controls (Figure 1a). Dark‐grown seedlings were not significantly different than non‐transformed controls (Figure 1a,b insets). Under blue light (Figure 1b), there was no significant difference between control and peptide‐expressing plants, while *phyB* did have a slight, but significant increase in relative hypocotyl length, consistent with previous observations (Neff and Chory, 1998).

### 3.2 Growth against a gravitational vector in red light

**Figure 2:**
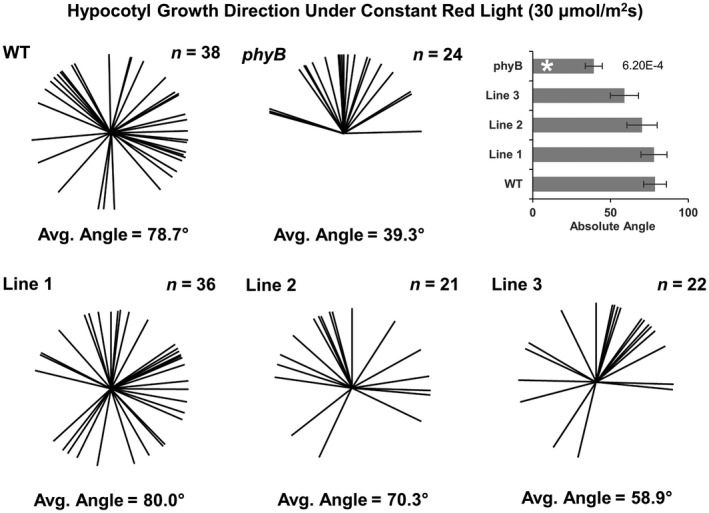
PEP6‐32 expressing plants exhibit normal directional growth under red light. Graphical depiction of non‐transformed control Col‐0 (WT), independent transformed lines expressing the PEP6‐32 peptide (Lines 1–3), and *phyB* plants after 96 hr of 30 μmol m^−2^ s^−1^ red light. Measurements taken from three independent experimental replicates. Sample size for each line is listed in the top right of each box. The average angle of growth is listed at the bottom of each box and is defined as the absolute angle from 0–180 where 0 is straight up (opposite the direction of gravity) and 180 is straight down (in the direction of gravity). Asterisk indicates statistically significant difference between transgenic seedlings and wild‐type seedlings using the Mann–Whitney *U* test. *p* Values are listed to the right of the bars for the significant results. Error bars indicate standard error of the mean

In addition to regulating hypocotyl elongation under red light, phyB also acts to guide directional growth of seedlings against the gravitational vector (Poppe, Hangarter, Sharrock, Nagy, & Schafer, 1996). Under constant red light, seedlings grow in various directions, not vertically. This phenotype is not observed in *phyB* seedlings, as they grow straight under red light. This has been shown to be caused by phyB's inhibition of PIFs which are involved in the development of the gravity sensing endodermal amyloplasts (Kim et al., 2011), and requires participation specific 14‐3‐3 proteins (Mayfield, Folta, Paul, & Ferl, 2007). To test the response in the PEP6‐32 expressing plants, seedlings were grown for 96‐hr length under constant red light (30 μmol m^−2^ s^−1^) and compared with *phyB* and wild‐type seedlings (Figure 2). For this experiment, the growth direction was measured from 0 to 180° with 0° being straight up and 180° being directly down, in line with the direction of gravity. As expected, the *phyB* mutants generally grew upright under constant red light with the average deviation of 39.3° from vertical. There was no significant difference in the angle for the peptide‐expressing plants when compared to WT controls.

### 3.3 Effect of PEP6‐32 on cotyledon expansion

**Figure 3:**
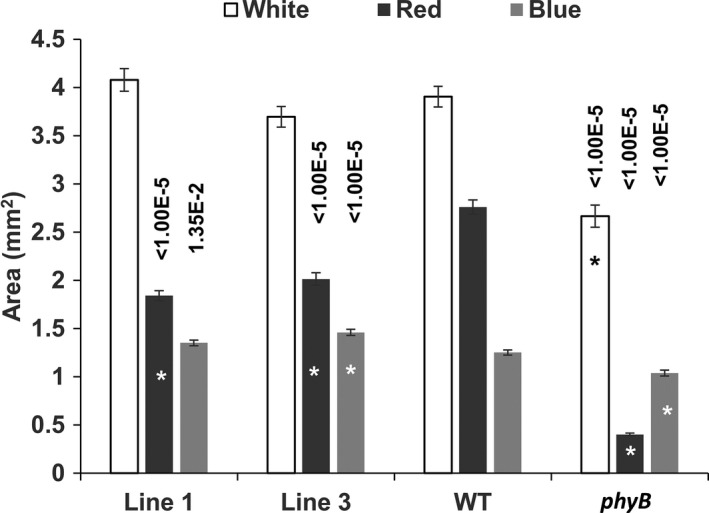
PEP6‐32 expressing plants exhibit smaller cotyledons under red light. Cotyledon size of a non‐transformed Col‐0 isoline (WT), independent transformed lines expressing the PEP6‐32 peptide (Lines 1 and 3), and *phyB* plants after 7 days under long‐day conditions. The fluence rate of white light was 96 μmol m^−2^ s^−1^, red light was 30 μmol m^−2^ s^−1^, and blue light was 10 μmol m^−2^ s^−1^. The results shown here are an average of two independent experiments, *N* = 73–82 per line. ImageJ was used to determine area of each cotyledon in mm^2^. Asterisks indicate statistically significant difference between transgenic or mutant seedlings compared with wild‐type seedlings using the Mann–Whitney *U* test. *p* Values are listed above the bars for the significant results. The same test also indicates that PEP6‐32 seedling cotyledon expansion is significantly different from *phyB* mutants (*p* < .00001). Error bars indicate standard error of the mean

Cotyledon expansion is also observed during photomorphogenic development in response to red light. This process has been shown to be controlled redundantly by both phytochromes (phyA and phyB) and cryptochrome (cry1) with phyB and cry1 the major components under white light, phyB under red light, and cry1 under blue light (Neff and Chory, 1998). Seedlings expressing *PEP6‐32* were grown under 100 μmol m^−2^ s^−1^ white light, 30 μmol m^−2^ s^−1^ red, or 10 μmol m^−2^ s^−1^ blue light with a 16‐hr photoperiod for 7 days. Afterward, the cotyledons were removed from the plants and imaged. The surface area was calculated using ImageJ. The results in Figure 3 show that under white or blue light *phyB* seedling cotyledons were smaller than wild type, yet the *PEP6‐32* lines were the same size or slightly larger. Under red conditions, the *PEP6‐32* lines showed a significantly decreased area, but not to the same extent as *phyB*. Interestingly, the cotyledon size of the two independent *PEP6‐32* lines was slightly, but significantly greater than non‐transformed controls under blue light, in contrast to *phyB* plants that are smaller than wild‐type seedlings.

### 3.4 Differences in red light‐regulated gene expression

**Figure 4:**
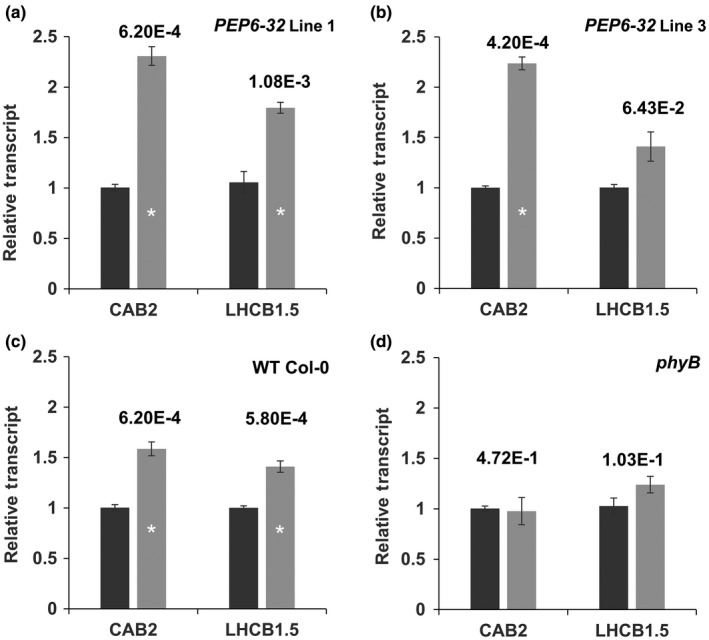
Expression of the peptide does not affect induction of *CAB2* or Lhcb1*5. Fold change in the steady‐state accumulation of *CAB2* and Lhcb1*5 transcripts in independent peptide‐expressing lines (a,b), compared with a non‐transformed control (Col‐0; c), and *phyB* mutant seedlings (d). Transcript levels were measured using RT‐qPCR after a 1 hr 8 μmol m^−2^ s^−1^ red light treatment of 4‐day‐old etiolated seedlings relative to dark‐grown controls. Results shown here are an average of three independent experimental replicates and three technical RT‐qPCR replicates. The *AtUFP* transcript was used as a constitutively expressed reference. Error bars indicate standard error of the mean. The asterisk indicates a statistically significant difference between transgenic and non‐transformed controls Mann–Whitney U test. P values are listed above the bars for the significant results

If the peptide interacts with phyB photosensor chemistry or signal transduction directly, changes in the timing or amplitude of phyB‐mediated transcript accumulation would be observed. Transcript accumulation corresponding to two red light‐regulated members of the *Light‐Harvesting, Chlorophyll‐Binding* (*Lhcb* or *Chlorophyll a/b Binding;* cab) gene family (*CAB2* and LHCB1*5) was monitored in response to a short red light treatment. Four‐day‐old etiolated seedlings were treated with 8 μmol m^−2^ s^−1^ of red light for 1 hr or kept in complete darkness as a control. The results show that there was no significant difference in accumulation of either of the two transcripts in the *phyB* mutant seedlings (Figure 4d), while wild‐type seedlings exhibited a typical and significant induction of both (Figure 4c). The *PEP6‐32* seedlings showed normal induction of both transcripts (Figure 4a,b) comparable to wild‐type, non‐transformed seedlings.

### 3.5 Peptide effect on the flowering time

**Figure 5:**
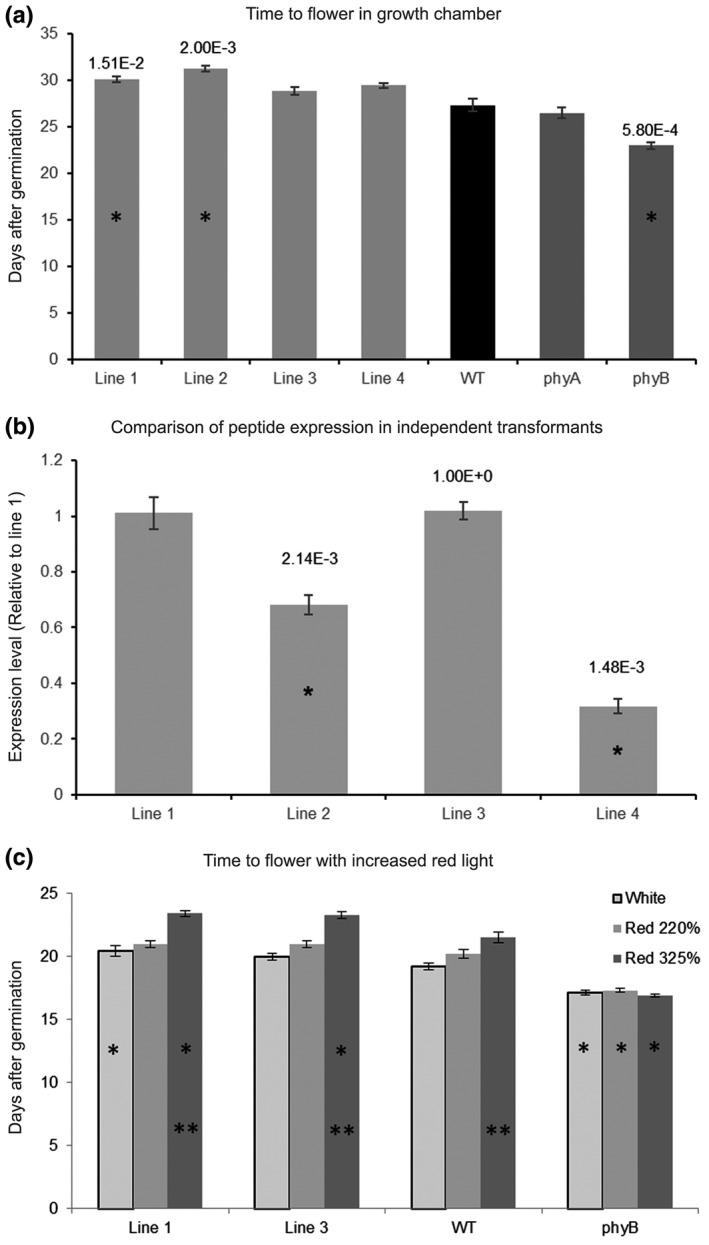
The peptide does not induce early flowering. (a) Time to flower of a non‐transformed Col‐0 isoline ( WT), independent transformed lines expressing the PEP6‐32 peptide (Lines 1–4), *phyA* plants, and *phyB* plants in a growth chamber at 21°C under long‐day conditions. The data indicate the average number of days until flowering. The asterisk indicates a statistically significant difference from wild‐type plants. *p* Values are listed above the bars for significant differences between PEP6‐32 transgenic seedlings and non‐transgenic controls. Control and transgenic seedlings were also significantly different from *phyB* mutants (*p* < .003). (b) Levels of PEP6‐32 transcript in the different independent transformed lines relative to Line 1, as measured using RT‐qPCR. Results shown here are an average of two independent RT‐qPCR experiments comprised of three technical replicates. The asterisk indicates a statistically significant difference compared with Line 1. All p values are listed above the bars. (c) The effect of increasing proportion of red light on flowering time. Lines 1, 3, WT, and *phyB* were grown in LED light chambers set to three conditions designed to mimic the light spectrum of the growth chamber. In the treatments, the proportion red light was increased to 220% or 325% of normal (see Figure S1). A single asterisk indicates statistically significant difference compared with wild‐type seedlings. The double asterisk indicates a statically significant difference from plants grown under normal white light for each respective line *p* ≤ .05. Transgenic PEP6‐32 seedlings and *phyA* mutants flowered significantly later than *phyB* mutants (*p* < .0004). Error bars indicate standard error of the mean

The phyB photoreceptor also has a central role in the regulation of flowering. To test whether the PEP6‐32 peptide affected phyB (or other) response during the floral transition, Col‐0, *phyA* (far‐red sensing impaired)*, phyB*, and segregating positive peptide and non‐transgenic seedlings were transplanted into pots 7 days after germination. The plants were grown in a growth chamber under long‐day conditions, and then, flowering was scored when the inflorescence was 1 cm in length or greater. The results show that instead of trending toward early flowering, the PEP6‐32 lines trend toward slightly later flowering (Figure 5a). The *phyB* mutant plants flowered about 4 days before Col‐0, but the peptide lines flowered 2–4 days later. Two PEP6‐32 expressing lines exhibited a short, but significantly later time to flower while two others did not. The corresponding mRNA levels were examined and indicate that steady‐state transcript levels did not correlate with the slight delays observed in the timing of flowering (Figure 5b).

Another way to examine the potential role of PEP6‐32 in phyB‐mediated signaling during flowering would be to monitor flowering time in relevant genotypes in red‐enriched light environments. Increasing the amount of red light should amount to increased photoactivated phyB and result in delayed flowering. For this experiment, plants were grown under LED light containing blue, green, amber, and red light (450, 530, 590, and 660 nm, respectively) and the same conditions with an increased the amount of red light (660nm) at the cost of amber light (590nm) to maintain constant photosynthetically active radiation (Supplemental Figure 1). Three sets of treatments were used, red, blue, green, and amber at 100 µmol m^−2^ s^−1^, the same treatment with 220% red, and the third treatment with 325% red. Under all three conditions, *phyB* mutant plants showed no change in the time to flower as expected (Figure 5c). At 325% red, both peptide‐expressing lines showed an increased time to flower which was significantly different from non‐transformed controls as well as different from the control white light grown plants. Increasing red to 220% had a slight, but not significant difference in time to flower for both WT and peptide‐expressing lines as compared to the control white light treatment. These data indicate that the peptide is not impairing phyB activity with respect to flowering time (Figure 5a,c).

### 3.6 Exogenous application of the peptide to non‐transformed plants

It was important to test whether a synthetic growth regulator like PEP6‐32 could modulate its effects when applied externally. To test this, a synthesized version of the peptide was added to solid and liquid media at relatively high concentrations and hypocotyl growth inhibition was measured after exposure to constant red light illumination. Fluence rate/response tests were performed to test the effect of exogenously applied peptide under these conditions.

**Figure 6:**
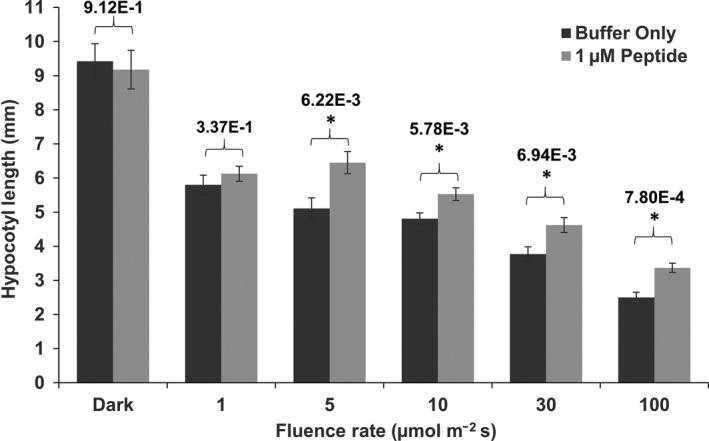
WT plants grown on media supplemented with synthesized PEP6‐32 exhibit a slight but significant increase in hypocotyl lengths under constant red light. Average hypocotyl length of non‐transformed seedlings grown on minimal media containing 1 μM of peptide (light bars) or a buffer control (dark bars) after 96 hr of 1, 5, 10, 30, or 100 μmol m^−2^ s^−1^ red light. Data were pooled from multiple independent experiments, *N* = 19–64. The asterisk indicates a statistically significant difference between plants grown in the presences of the PEP6‐32 peptide compared with media without the peptide, using Mann–Whitney *U* test. All *p* values are listed above the bars. Error bars indicate standard error of the mean

The synthesized peptide was added to buffer at 1 μM in solid minimal media. The seedlings were grown vertically under 1, 5, 10, 30, or 100 μmol m^−2^ s^−1^ of constant red light for 96 hr. The results reveal that plants grown in the presence of PEP6‐32 exhibited in a slight yet significant difference in hypocotyl length at fluence rates of 5 μmol m^−2^ s^−1^ or greater (Figure 6). No significant difference was observed in dark‐grown plants or those grown under 1 μmol m^−2^ s^−1^ red light.

## 4 DISCUSSION

The 2017 report by Bao and colleagues provided evidence that new synthetic regulators of plant growth and development could be identified within populations of transgenic plants expressing random DNA sequences. Hundreds of reproducible phenotypes have been observed in plants from these populations, and it is now the challenge to identify the mechanisms by which the peptides (or possibly their nascent RNAs) exert their effects on specific aspects of plant biology. The report showed that expression of the *PEP6‐32* sequence led to phenotypes that generally mimic those of a partial *phyB* mutant under red light. Hypocotyl growth inhibition was impaired consistent with a role for the peptide in attenuating phyB sensing, signal transduction, or response.

This observation provided an excellent starting point to examine the precise mechanism of the peptide's effect, because red light effects on plant development and gene expression are well described, both genetically and physiologically. Red light responses are typically mediated by phytochrome B (phyB). The receptor is activated, translocated, and accumulates in the nucleus within several hours of illumination (Gil et al., 2000; Yamaguchi, Nakamura, Mochizuki, Kay, & Nagatani, 1999), where it affects gene expression (Tepperman et al., 2004). Mechanistically, phyB directs various nuclear red light‐regulated responses through interaction with Phytochrome Interacting Factors (PIFs) (Huq et al., 2004; Huq & Quail, 2002; Kim et al., 2003; Ni, Tepperman, & Quail, 1999). The light‐activated phyB receptor binds to PIFs, in some cases preventing their association with promoter sequences in target genes (Huq & Quail, 2002) while controlling the proteolytic degradation of others (Bauer et al., 2004). The intricate coordination of signaling steps, re‐localization of proteins, DNA binding, protein turnover, then integration into hormonal response, changes in turgor and cell wall plasticity present many opportunities for interference from a rogue chemistry. Precise characterization of the localization and timing of PEP6‐32's effect on plant development would potentially provide a starting point for discovery of interactors, as well as a basis to further describe detailed facets of red light response. Therefore, we tested the hypothesis that the synthetic peptide PEP6‐32 interfered with some aspect of phyB‐mediated signaling.

The hypothesis was tested by examining a set of well‐characterized phyB‐mediated responses, and the results indicate that the PEP6‐32 peptide is playing a role in discrete tissues that may or may not be directly connected to phyB response. Hypocotyl growth inhibition and cotyledon expansion were clearly impaired during red light‐mediated development, yet less so under blue or white light (Figures 1 and 3). These results support the hypothesis that the response is related to phyB.

However, examination of other classical phyB‐mediated responses does not support the hypothesis. Wild‐type seedlings grown under red light on vertical plates exhibit directional growth abnormalities, as hypocotyls grow in various directions relative to the gravitational vector. However, *phyB* mutants grow straight up on vertical plates in red light (Kim et al., 2011; Poppe et al., 1996). Seedlings expressing *PEP6‐32* do not show a significantly different phenotype relative to non‐transgenic controls (Figure 2), so while some facets of early red light signaling are impaired, others are completely unaffected. This finding indicates that the effect is not likely occurring at the level of the receptor or primary signaling events or that thpe directional growth effect develops separately from stem growth inhibition and cotyledon expansion where the effect of the peptide is observed.

It is also well established that specific transcripts are induced by a short, single red light treatment in etiolated seedlings, under the direction of phyB (Kuno, Muramatsu, Hamazato, & Furuya, 2000; Tepperman et al., 2004). Red light treatments induce steady‐state transcript accumulation from members of the *Light‐Harvesting, Chlorophyll‐Binding* (*Lhcb*, also *cab*) gene family (Karlin‐Neumann, Sun, & Tobin, 1988; Kaufman, Thompson, & Briggs, 1984). The seedlings expressing PEP6‐32 show normal transcript accumulation in response to red light treatment. These results also indicate that PEP6‐32 is not likely participating in red light signaling or integration, again not supporting the hypothesis of direct connections with phyB signal integration.

The effects on long‐term phyB‐mediated responses were also examined. Flowering is controlled by a variety of signals meant to assess plant age, season, local growing environment, and other factors that may impact seed production and viability (Putterill et al., 2004). In Arabidopsis, these signals primarily control flowering through the suppression or promotion of CONSTANS (CO) and FLOWERING LOCUS T (FT) expression and activity (Valverde et al., 2004). Red wavebands delay flowering by promoting CO degradation through a COP1‐independent pathway mediated by phyB (Demotes‐Mainard et al., 2016). Consequently, in *phyB* mutants, CO remains elevated, increasing expression of FT which can then induce flowering (Endo, Nakamura, Araki, Mochizuki, & Nagatani, 2005). Therefore, *phyB* mutant plants flower early. If the PEP6‐32 had a role in phyB signaling later in development, seedlings would be expected to flower early, phenocopying *phyB* mutants. However, the plants expressing the peptide flowered at the same time or even slightly later than wild‐type plants, which is exactly the opposite of what occurs in *phyB* mutants. The effect is minor yet statistically significant in some transgenic lines. The result is important in that it indicates that the effect of PEP6‐32 in limiting red light response is likely limited to early seedling growth and is not exerting an impairment of all red light‐related responses.

Taken together, the interpretation is that PEP6‐32 is affecting cell expansion in the developing seedling, conditionally under red light. Hypocotyl growth rate inhibition and cotyledon expansion are repressed, two processes that are dependent on cell expansion, yet in opposing directions. Cotyledons typically expand upon illumination, yet here are smaller in the presence of the peptide after red light treatment. Hypocotyls are longer in the presence of the peptide when their elongation should be limited. It is tempting to speculate that the peptide could be functioning near the apical meristem, possibly by interacting with the hormone synthesis, transport, or sensitivity that changes the location of expansion from the hypocotyl to cotyledon during the transition from darkness to light. The response is specific to red light, where the same expansion/inhibition patterns have been shown to be regulated in the same way by blue or far‐red light. It is unclear why the effect is only observed under red light. One possibility is that blue and far‐red signals excite alternative mechanisms that mask the red light response.

The inhibition of red light seedling response was also induced by application of exogenous peptide. Seedlings grown on media containing the peptide exhibited longer hypocotyls. The effects were less pronounced in magnitude compared with transgenic seedlings expressing PEP6‐32, but exhibited statistically separable, dose‐dependent action over two concentrations. These findings confirm that the phenotype is caused by the peptide and not another product of the transgene, such as an mRNA. Furthermore, this shows that the peptide can be taken up by seedlings to induce a phenotypic change.

The seminal work by Bao et al., (2017), illustrated that biologically active synthetic peptides may be identified by studying aberrancies in populations of random DNA‐expressing plants. This report expands these findings to characterize the effect of the PEP6‐32 peptide, showing effects isolated to specific developmental windows, tissues, and conditions, as well as effects induced by exogenous application. Future experiments will examine the structure‐function aspects of the amino acid sequence to further probe biochemical activity and will test for specific protein–protein interactions. The trials presented in this report show that synthetic peptides created from random DNA sequence have the capacity to produce new molecules with discrete connections to plant biology. The results further validate this approach as a way to discover novel synthetic chemistries, as well as provide new tools to explore basic questions in plant growth, development, and metabolism.

## CONFLICT OF INTEREST STATEMENT

The authors declare no conflict of interest associated with the work described in this manuscript.

## AUTHOR CONTRIBUTIONS

T.S. performed all experiments that produced data for this report and provided the first draft of the manuscript; R.C. identified and isolated the original plant line studied; Z.B. participated in generation of plant materials and supervision of the experiments; M.C. designed and prepared the constructs for transformation and developed the random peptide‐expressing lines; K.F. conceived the project and wrote the article with contributions from all of the authors and agrees to serve as the author responsible for contact and ensures communication.
