# Supplementary figures and images for: A synthetic peptide encoded by a random DNA sequence inhibits discrete red light responses
Source: Plant Direct. 2019 Oct 14;3(10):e00170. doi: 10.1002/pld3.170 (PMC6790650; doi:10.1002/pld3.170)

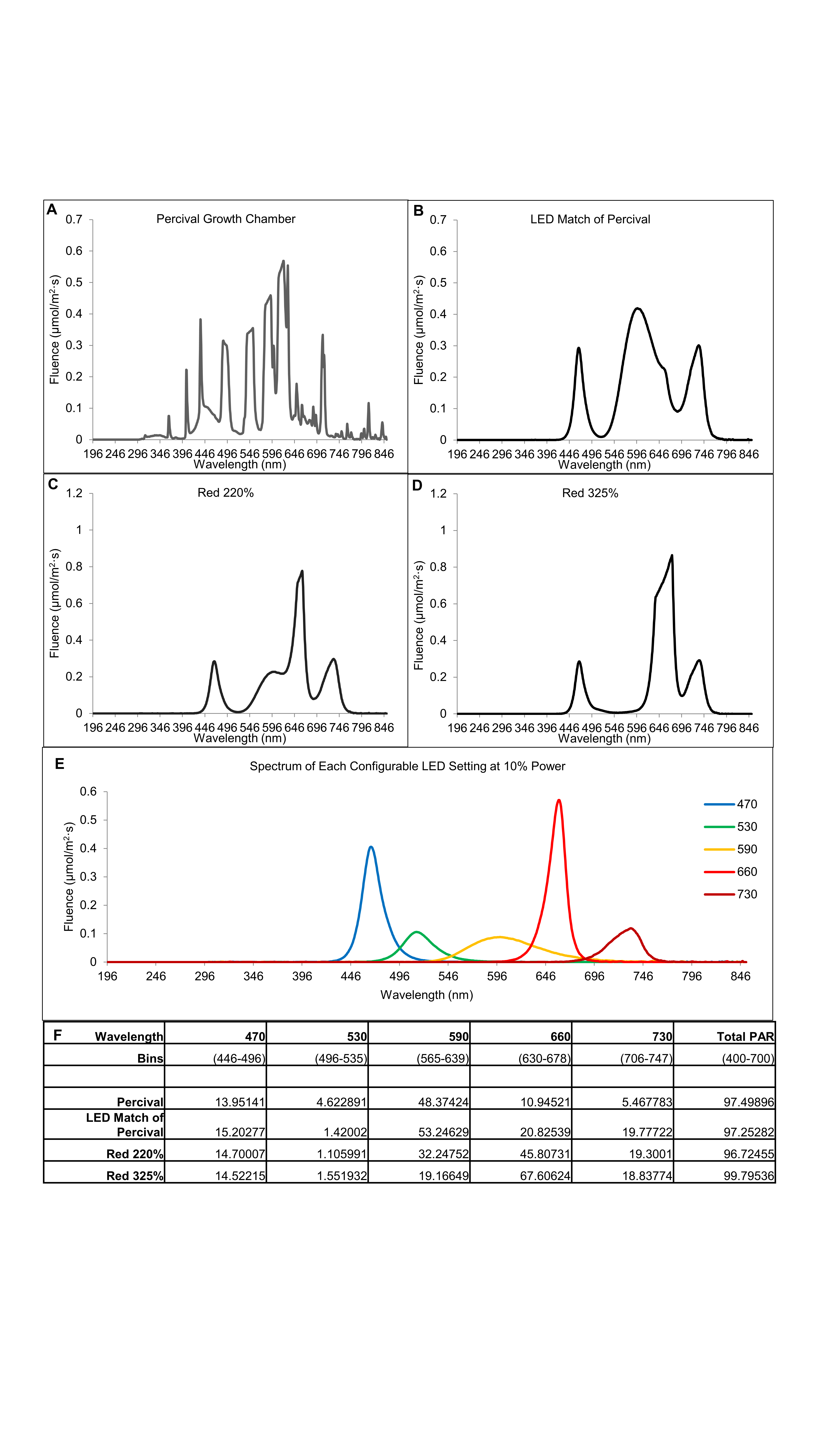

Supplement: Supplementary file 1 [file PLD3-3-e00170-s001.PNG]

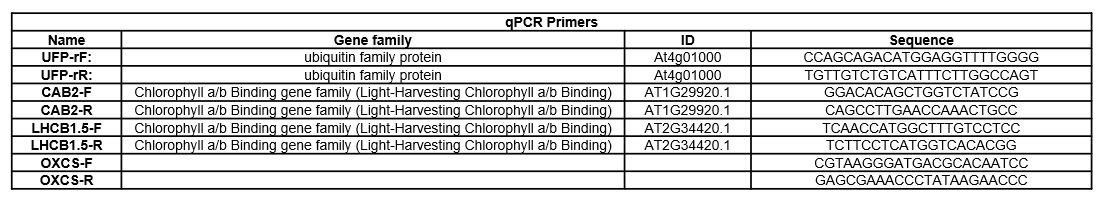

Supplement: Supplementary file 2 [file PLD3-3-e00170-s002.JPG]
